# Effects of Early Initiation of Polymyxin B Hemoperfusion Therapy in Patients with Cancer with Refractory Septic Shock

**DOI:** 10.3390/jcm13041009

**Published:** 2024-02-09

**Authors:** Jae Hoon Lee, Won Ho Han, Hyun-jae Im, Jee Hee Kim

**Affiliations:** 1 Critical Care Medicine, National Cancer Center, 323 Ilsan-ro, Ilsandong-gu, Goyang-si 10408, Republic of Korea; 52223@ncc.re.kr (J.H.L.); neptunelight88@gmail.com (H.-j.I.); 2 Uijeongbu Eulji Medical Center, Eulji University School of Medicine, Uijeongbu 11759, Republic of Korea; anesth67@gmail.com

**Keywords:** sepsis, in-hospital mortality, risk factors, hemodynamics, lactate clearance, organ failure

## Abstract

**Background**: We aimed to analyze the correlation between in-hospital mortality and hemodynamic changes, using polymyxin B-immobilized fiber column direct hemoperfusion (PMX-DHP) initiation time in patients with cancer with refractory septic shock. **Methods**: Forty-six patients with cancer who received PMX-DHP for refractory septic shock were retrospectively analyzed and classified into early (≤3 h between refractory septic shock and PMX-DHP; *n* = 17) and late (>3 h; *n* = 29) initiation groups. The vasopressor inotropic score (*VIS*), sequential organ failure assessment (SOFA) score, and lactate clearance before and 24 h post-PMX-DHP were compared. **Results**: Overall, 52.17% died from multiple organ dysfunction, with a lower mortality rate in the early initiation group. The *VIS* and SOFA score decreased in both groups, but the magnitude of decrease was not significant. Lactate clearance improved in both groups, with greater improvement in the early initiation group. Univariable analysis identified associations of in-hospital mortality with early initiation, ΔC-reactive protein, lactate clearance, ΔSOFA score, and Δ*VIS*. Multivariable analysis demonstrated associations of in-hospital mortality risk with ΔSOFA score and early PMX-DHP initiation. Overall survival was higher in the early initiation group. Early initiation of PMX-DHP in patients with cancer with refractory septic shock reduced in-hospital mortality and improved lactate clearance.

## 1. Introduction

Sepsis induces life-threatening organ dysfunction through the dysregulation of host responses to infection [1]. Globally, sepsis is fatal for 11 million of the 49 million people who contract it each year, equivalent to a total global mortality rate of 19.7%. Sepsis can progress to septic shock, characterized by the necessity for vasopressors to address hypotension and lactic acidosis, with a case fatality rate of 30–40% [2,3]. Early resuscitation is crucial for improving multiple organ dysfunction and reducing mortality in patients with septic shock [4]. This involves administering an initial fixed volume of 30 mL of intravenous crystalloid fluid per kilogram of body weight and maintaining a mean arterial pressure (MAP) of 60–65 mmHg with adequate vasopressors [5,6]. Additionally, echocardiography, arterial and central venous catheterization, pulmonary artery catheterization, hemodynamic monitoring, and measurement of inferior vena cava variation and lactate levels are important for managing septic shock [6,7,8]. With advancements in early detection and treatment strategies, a declining trend in sepsis-related mortality has been observed [8]. However, sepsis remains a significant risk factor, particularly in immunocompromised patients with cancer undergoing treatment, as well as in those with a confirmed poor prognosis. Of note, the incidence of sepsis is 3 to 5 times higher in patients with cancer, at 16.4 cases per 1000 persons, compared to those without cancer. Furthermore, the sepsis-related mortality rate is approximately 3-fold higher than the annual cancer death rate (10% vs. 37.8%), although mortality rates vary across cancer types [8].

Polymyxin B-immobilized fiber column direct hemoperfusion (PMX-DHP) is a potential therapy developed in 1994 to reduce endotoxin levels and treat septic shock; it effectively enhances hemodynamics and respiratory function in patients with septic shock caused by intra-abdominal infection [9]. However, whether it contributes to survival and prevents organ failure is controversial, and there are no established guidelines on the best time to perform PMX-DHP [10,11,12,13].

To date, studies on PMX-DHP have mainly targeted patients without cancer, resulting in a scarcity of literature on patients with cancer. This prompted the present investigation, in which we analyzed the correlation between PMX-DHP initiation time and in-hospital mortality in patients with cancer with refractory septic shock. Additionally, we aimed to verify whether hemodynamics, sequential organ failure assessment (SOFA) scores, and lactate clearance improve following PMX-DHP treatment.

## 2. Materials and Methods

This single-center, retrospective study was conducted in 46 patients with cancer, aged ≥18 years, who received PMX-DHP to treat refractory septic shock at the intensive care unit of our institution between October 2019 and August 2023. Septic shock was identified based on the criteria established for identification of sepsis and septic shock (Sepsis-3), including the requirement of vasopressors to maintain a MAP of ≥65 mm Hg and a serum lactate level of >2 mmol/L in the absence of hypovolemia [1]. PMX-DHP was administered to patients who voluntarily consented to the associated treatment cost. Refractory septic shock was defined by the presence of hyperlactatemia (>2 mmol/L (18 mg/dL)) and an ongoing need for ≥0.5 μg/kg/min of norepinephrine or additional dose of vasopressin to maintain a MAP of >65 mmHg, even after adequate volume resuscitation (at least 30 mL/kg of intravenous crystalloid fluid within the first 3 h) [4,14]. We administered intravenous hydrocortisone at a dose of 200 mg/d, provided as 50 mg intravenously every 6 h, to all patients diagnosed with refractory septic shock. Abdominal infection was characterized as peritoneal inflammation in response to microbial invasion, resulting in purulence within the peritoneal cavity. It includes conditions such as peritonitis, diverticulitis, cholecystitis, cholangitis, and pancreatitis. In contrast, non-abdominal infections include those originating outside the abdominal cavity, such as pneumonia, bloodstream infection, genitourinary infection, and infections related to vascular catheters [15]. Laboratory analysis and blood culture tests were conducted for all patients before and after treatment. Broad-spectrum antibiotics were administered to cover bacterial strains adequately, considering previous blood culture results and antibiotic susceptibility.

Polymyxin B fiber (Toraymyxin^®^ Cartridge, Toray Medical, Tokyo, Japan) was used as an adsorbent in this study. The binding site of polymyxin B on the endotoxin is the lipid A portion, involving both ionic and hydrophobic interactions. Lipid A is the toxic component of the endotoxin and exhibits a highly conserved structure across various Gram-negative bacterial species and strains. As such, using polymyxin B as a ligand to effectively bind various types of endotoxins in Gram-negative sepsis is a logical and reasonable approach [16]. For PMX-DHP, the blood flow rate was set at 100 mL/min, and anticoagulation therapy was initiated using nafamostat mesylate, a synthetic serine protease inhibitor, at a rate of 20 mg/h. Notably, nafamostat mesylate, widely employed in Japan for blood purification, has demonstrated minimal impact on bleeding risk compared to unfractionated heparin or a non-anticoagulant [17,18,19]. In our study, patients faced a heightened risk of thrombocytopenia and coagulopathy due to refractory septic shock [20,21]. Consequently, we opted for nafamostat mesylate as an anticoagulant, considering its relatively low potential for bleeding. The anticoagulation regimen was adjusted to a rate of 20–40 mg/h based on the patient’s platelet count and bleeding tendency. The duration of PMX-DHP was set as one cycle lasting 12 h [22,23]. If PMX-DHP treatment was temporarily halted for operation or interventional therapy, it was resumed upon completion of these procedures. Additional cycles were administered if elevated vasopressor requirements indicated ongoing organ failure even after the initial cycle. Patients exhibiting unstable vital signs during treatment, making further therapy impractical, had PMX-DHP discontinued. Those who received less than 2 h of treatment or died during the course of treatment and expressed a do-not-resuscitate directive or a desire to discontinue life-sustaining treatment after diagnosis were excluded. Patients who received PMX-DHP treatment within 3 h from the diagnosis of refractory septic shock were assigned to the “early initiation” group, while those who were treated after 3 h or more had elapsed since their diagnosis were assigned to the “late initiation” group.

The following are patient characteristics entered as variables for analysis: age, sex, underlying disease (diabetes mellitus, hypertension, cardiovascular disease, chronic kidney disease, chronic obstructive pulmonary disease), cancer type (genitourinary, abdominal, others), parameters before and after PMX-DHP (white blood cell count, presence of neutropenia, hemoglobin level, platelet count, C-reactive protein [CRP] level, lactic acid level, international normalized ratio [INR], ratio of arterial oxygen partial pressure to fraction of inspired oxygen [P/F ratio]), Acute Physiology and Chronic Health Evaluation III (APACHE III) score, SOFA score, PMX-DHP frequency, PMX-DHP duration, PMX-DHP initiation time, transfusion, vasoactive inotrope score (*VIS*), primary infection site (abdominal, non-abdominal), microbiological culture, mechanical ventilator use, continuous renal replacement therapy (CRRT), source control methods (operation, interventional), and in-hospital mortality.

*VIS* was measured as follows [24]:

$$\begin{array}{l} VIS=dopamine\;dose\;(µg/kg/min)+dobutamine\;dose\;(µg/kg/min)\\ \;+100\times epinephrine\;dose\;(µg/kg/min)\\ \;+10\times milrinone\;dose\;(µg/kg/min)\\ \;+10,000\times vasopressin\;dose\;(units/kg/min)\\ \;+100\times norepinephrine\;dose\;(µg/kg/min) \end{array}$$

Lactate clearance was determined as follows [25]:

$$Lactate\;Clearance=\frac{\left({Lactate}_{Initial}-{Lactate}_{Delayed}\right)}{{Lactate}_{Initial}}\times 100(\%)$$

Using patient data, correlations of the aforementioned variables with PMX-DHP initiation time (early initiation group ≤ 3 h; late initiation group > 3 h) were assessed by calculating differences in values reported before and 24 h after PMX-DHP. This study was approved by the Institutional Review Board of the National Cancer Center (Approval No.: NCC2023-0170). The data access period for the manuscript was from 3 July 2023, the date of IRB approval, to 31 October 2023. The requirement for the acquisition of informed consent from patients was waived owing to the retrospective nature of this study.

### Statistical Analysis

Categorical variables were compared between groups using the Pearson χ^2^ test and Fisher’s exact test, and continuous variables were compared using *t*-tests for parametric data and the Wilcoxon rank sum test for nonparametric data. Continuous variables are expressed as mean ± standard deviation or median (minimum–maximum or range). Statistical significance was set at *p* < 0.05. To determine the optimal initiation time of treatment, we utilized the mean time from a prior large-scale RCT as a reference [12]. Subsequently, the log-rank statistic method was performed to establish an optimal cut-off point.

To analyze differences before and after PMX-DHP treatment between the two groups, laboratory data, SOFA score, and *VIS* were compared. Due to the limited sample size and non-normal distribution, the Wilcoxon signed-rank test was conducted. Subsequently, clinical parameters before and after treatment were compared between the two groups. Logistic regression analysis was performed to identify factors influencing in-hospital mortality. Initially, univariable analysis was conducted for each variable, and the final variable set for the multivariable model was selected through backward elimination in the univariable analysis with a threshold *p*-value < 0.05. Only variables demonstrating sufficient adjusted significance in predicting in-hospital mortality were included in the final model. Odds ratios (ORs) and 95% confidence intervals (CIs) were calculated for each variable. Kaplan–Meier analysis was conducted to compare in-hospital mortality between the early and late initiation groups. All statistical analyses were performed using SAS^®^ version 9.4 for Windows^®^ (SAS Institute, Cary, NC, USA).

## 3. Results

### 3.1. Characteristics of the Patients Undergoing PMX-DHP Treatment

Forty-six patients received PMX-DHP for refractory septic shock. The mean age of the patients was 64.78 years, and 24 (52.17%) died during the hospitalization period due to multi-organ failure caused by septic shock. 

Among the 46 patients, 17 underwent PMX-DHP for refractory septic shock within 3 h of diagnosis, while 29 initiated PMX-DHP after 3 h of diagnosis. The predominant cause of late initiation was delayed decision making by caregivers (44.83%), followed by the implementation of additional procedures (operation or intervention, 31.03%) and delayed determination of treatment plans by the primary medical department (24.14%).

The most frequent cancer type was genitourinary (54.35%), followed by abdominal (30.43%). The most frequent underlying disease was hypertension (47.83%), followed by diabetes mellitus (29%). PMX-DHP was predominantly performed as a single cycle in most patients (84.78%), with a median application time of 12 h (2.08–68.83). The median time to initiation of treatment was 5 h (0.6–27). The mean APACHE III score before PMX-DHP was 93.54 ± 28.89, while the mean SOFA score was 15.34 ± 3.34. The most frequent primary infection site was the abdomen (69.56%), and 89.13% of patients had confirmed bacteremia based on blood culture results. The median *VIS* of patients maintaining a MAP of >65 mmHg was 242.50 (12–913). In pre-treatment laboratory data, the median hemoglobin level was 9.65 g/dL (7.2–17.8), platelet count was 103 × 10^3^/μL (2–352), and INR was 1.65 (1.01–6.11). A total of 84.78% of patients received transfusions. The mean units administered were 2.5 ± 2.5 packs for red blood cells, 1.26 ± 1.50 packs for plateletpheresis, and 4.87 ± 6.20 packs for fresh frozen plasma. Treatment for infection source control was performed in 56% of patients, excluding those with inadequate hemodynamic stability for operation or interventional therapy. Surgical treatment was administered to 36.96%, while 26.09% underwent interventional therapy. The majority of patients (91.30%) underwent mechanical ventilation, with an average duration of 5.83 ± 4.80 d. CRRT was applied in 80.43% of patients (Table 1).

Seventeen and twenty-nine patients were assigned to the early and late initiation groups, respectively. There were no differences in underlying disease and cancer types between the groups. Disease severity indices (mean APACHE III and SOFA scores) did not exhibit significant differences between the early and late initiation groups (APACHE III score: 92.12 ± 28.98 vs. 94.38 ± 29.32, *p* = 0.801; SOFA score: 14.88 ± 3.22 vs. 15.62 ± 3.44, *p* = 0.476). The median application time of PMX-DHP was 12 h for both groups, and there was no difference between the two groups (*p* = 0.212). The time to treatment initiation was 2.12 h (0.6–3) in the early initiation group and 6.92 h (3.22–27) in the late initiation group. The platelet count was higher in the early initiation group than in the late initiation group (113.00 (15–352) vs. 88.00 (2–235); *p* = 0.041). There were no differences in the results of other blood tests, microbiological culture, and the amount of transfusion between the two groups. The *VIS* before treatment showed no difference between the groups (236 (14–736) vs. 246 (12–913), *p* = 0.973). There were no differences in the treatment for infection source control and treatment methods (operation, interventional), mechanical ventilation use (100% vs. 86.21%, *p* = 0.281), duration of ventilator use (4.48 ± 3.51 days vs. 7.58 ± 4.55 days, *p* = 0.080), and CRRT (*p* = 0.258) between the two groups. In-hospital mortality was lower in the early initiation group than in the late initiation group (23.53% vs. 68.97%, *p* = 0.008) (Table 1).

### 3.2. Overall Survival (Early vs. Late Initiation)

**Figure 1:**
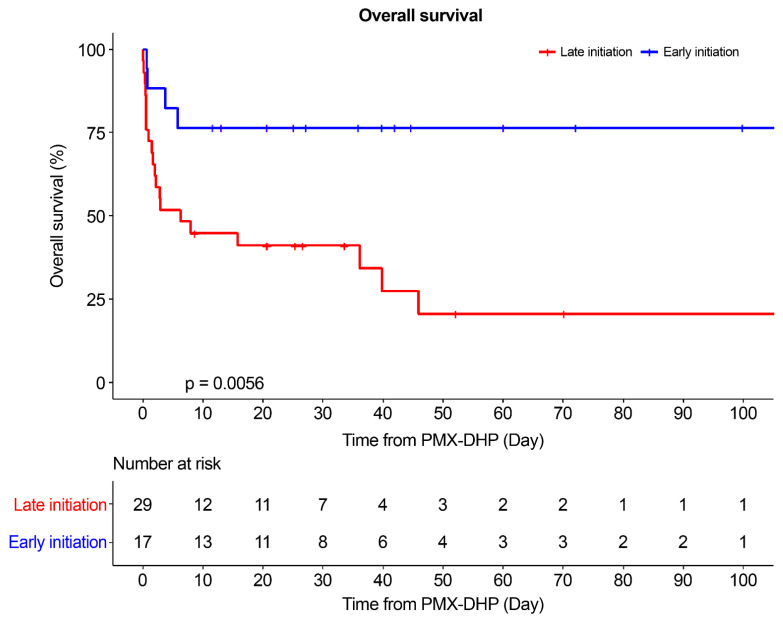
Kaplan–Meier survival analysis based on the initiation time of PMX-DHP in patients with refractory septic shock. The cumulative survival rate was significantly higher in the early initiation group than in the late initiation group (*p* = 0.0056). The log-rank test was used to compare survival curves. PMX-DHP: polymyxin B-immobilized fiber column direct hemoperfusion.

The number of deaths during the hospitalization period from the initiation of PMX-DHP was compared for both groups; four deaths were recorded in the early initiation group and twenty in the late initiation group (Table 1). The survival rate in the early initiation group was 76.47%, compared with 31.03% in the late initiation group, with a median survival period of 36.2 days. Meanwhile, the overall survival in the hospitalization period was higher in the early initiation group (*p* = 0.0056; Figure 1).

### 3.3. Changes in the Values of Variables after PMX-DHP Treatment (Early vs. Late Initiation)

**Figure 2:**
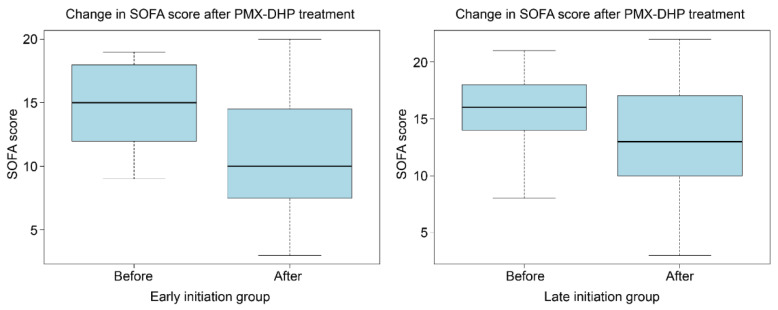
Changes in SOFA score associated with PMX-DHP initiation. The boxplots illustrate the changes in SOFA score after PMX-DHP treatment. In the early initiation group, the SOFA score decreased from 15.00 to 10.00 (*p* = 0.024), while in the late initiation group, it decreased from 16.00 to 13.00 (*p* = 0.009). SOFA: sequential organ failure assessment; PMX-DHP: polymyxin B-immobilized fiber column direct hemoperfusion.

**Figure 3:**
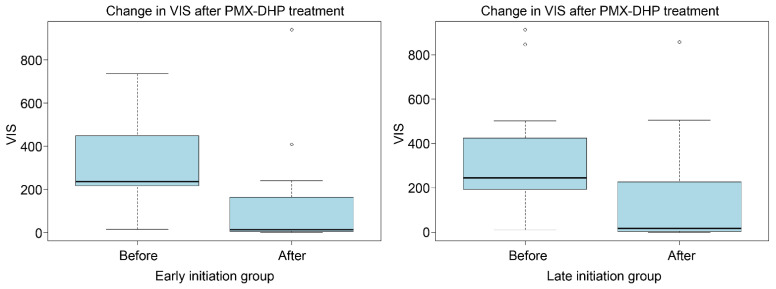
Changes in *VIS* associated with PMX-DHP initiation. The boxplots illustrate the changes in *VIS* after PMX-DHP treatment. In the early initiation group, the score decreased from 236.0 to 12.5 (*p* = 0.011), while in the late initiation group, it decreased from 246.0 to 18.0 (*p* < 0.001). PMX-DHP: polymyxin B-immobilized fiber column direct hemoperfusion; *VIS*: vasoactive inotrope score = (dopamine dose (µg/kg/min) + dobutamine dose (µg/kg/min) + 100 × epinephrine dose (µg/kg/min) + 10 × milrinone dose (µg/kg/min) + 10,000 × vasopressin dose (units/kg/min) + 100 × norepinephrine dose (µg/kg/min).

A comparison of variables before and 24 h after PMX-DHP showed that, in both the early and late initiation groups, *VIS* (*p* = 0.011 vs. *p* < 0.001) and SOFA scores (*p* = 0.024 vs. *p* = 0.009) significantly decreased after PMX-DHP (Table 2; Figure 2 and Figure 3).

The platelet count decreased in the early initiation group after PMX-DHP treatment (*p* < 0.001). However, there was no significant change in the late initiation group (*p* = 0.115). The CRP level increased in the early initiation group after PMX-DHP (*p* = 0.016). There was no significant change in the late initiation group (*p* = 0.687) (Table 2).

Subsequently, we compared changes in variables from before to after PMX-DHP between both groups. The hemoglobin level decreased slightly in both groups after treatment, with no significant difference between the two groups (*p* = 0.759). Platelet counts decreased in both groups, and the early initiation group exhibited a greater reduction than the late initiation group (−77.0 (−209–20) vs. −16.0 (−208–76); *p* = 0.017). The CRP level increased in the early initiation group and decreased in the late initiation group (1.50 (−2.2–23.97) vs. −0.24 (−12.76–18.48); *p* = 0.025). Lactate clearance improved in both groups, with the early initiation group showing greater improvement than the late initiation group (74.82 (−22.92–94.62) vs. 2.86 (−527.25–72.43); *p* < 0.001). The P/F ratio increased in both groups with no significant difference (*p* = 0.237). The SOFA score and *VIS* decreased in both groups, but there were no significant differences between the two groups (SOFA score; *p* = 0.400, *VIS*; *p* = 0.575) (Table 3).

### 3.4. Investigating Factors Influencing In-Hospital Mortality Using Univariable and Multivariable Analyses

Our univariable analysis of in-hospital mortality in patients with cancer with refractory septic shock after PMX-DHP showed that early initiation (OR = 0.139; 95% CI = 0.032–0.509; *p* = 0.005), the difference in CRP level before and after treatment (OR = 0.774; 95% CI = 0.610–0.902; *p* = 0.008), lactate clearance (OR = 0.990; 95% CI = 0.980–0.997; *p* = 0.018), the difference in SOFA score (OR = 1.460; 95% CI = 1.197–1.931; *p* = 0.001), and the difference in *VIS* (OR = 1.007; 95% CI = 1.002–1.013; *p* = 0.012) were correlated with in-hospital mortality. A multivariable analysis, utilizing the results of the univariable analysis, revealed that in-hospital mortality was associated with the difference in SOFA score before and after PMX-DHP (OR = 1.616; 95% CI = 1.204–2.170; *p* = 0.001) and early initiation (OR = 0.033; 95% CI = 0.002–0.462; *p* = 0.011; Table 4).

## 4. Discussion

This study was conducted to analyze the correlation between the initiation time of PMX-DHP and in-hospital mortality in patients with cancer with refractory septic shock and to verify whether hemodynamics and SOFA score improve after PMX-DHP. In this study, a mortality rate of 52.17% was observed in patients with refractory septic shock. Notably, there were limited direct comparisons of mortality rates in studies focusing on refractory septic shock in cancer patients. Consequently, we cited a previous meta-analysis of sepsis treatment in cancer patients, which reported a mortality rate of 48–62%, as a pertinent point of comparison. The observed mortality rate in our study did not show a significant difference when compared to the range reported in the aforementioned meta-analysis [26]. However, the earlier reported mortality has been found to be slightly higher than the mortality related to septic shock observed in non-hospitalized patients with cancer who received PMX-DHP (19–37.7%) [12,13,27]. This could be because the patients in our sample, unlike those in other studies, had an immunosuppressed status and showed a higher disease severity (APACHE III score; SOFA score) than those in studies performed at other centers. Furthermore, since the frequency of non-abdominal infections was relatively high in our study (32.61%), it could have contributed to a relatively increased mortality rate. Another possible explanation could be that the treatment initiation in the present study occurred at a comparatively more severe stage, as the initiation of PMX-DHP followed the diagnosis of refractory septic shock, than that in previous studies.

The result of this study suggested that the change in SOFA score after PMX-DHP is associated with an increase of in-hospital mortality. Additionally, the trend of the SOFA score was confirmed as a better predictor of mortality than the initial SOFA score. The SOFA score is an indicator used to assess and monitor organ dysfunction. According to previous research findings, the SOFA score is correlated with mortality, with particular emphasis on the trend of the SOFA score being associated with in-hospital mortality [28]. In this study, the SOFA score decreased in both groups, with a slightly greater change in the early initiation group than in the late initiation group (−4.00 ± 5.87 vs. −2.64 ± 4.37; *p* = 0.400). Therefore, the early application of PMX-DHP may have the potential to prevent multi-organ failure and reduce in-hospital mortality.

Vasopressors are essential for circulatory support in patients with septic shock. However, they can induce circulatory impairment and lead to complications such as tachyarrhythmia and digital ischemia, potentially increasing in-hospital mortality and length of stay [29,30]. Previous research has established a correlation between the *VIS* and in-hospital mortality. Furthermore, a reduction in the *VIS* has been found to be associated with improved in-hospital survival [31,32]. In our study, the requirement for vasopressors to maintain hemodynamics after PMX-DHP decreased in both groups. While the early initiation group showed a slightly greater reduction, the difference between the two groups was not significant (*p* = 0.575). Previous studies mostly reported relatively low vasopressor requirements. However, our study focused on patients with refractory septic shock, resulting in a notably higher initial *VIS* than that in other studies. Therefore, the study diverged from previous findings, considering that it was conducted in patients at a severe stage of septic shock. Additionally, since the SOFA score includes hemodynamic parameters, considering various factors contributing to organ failure may be more relevant for predicting mortality than solely assessing vasopressor requirements.

Lactate levels are commonly used as a biomarker for assessing tissue hypoperfusion and the severity of sepsis and predicting mortality in cases of septic shock [1,33]. Moreover, lactate clearance functions as an indicator of treatment response and can be used to predict mortality. Certain studies have indicated a significant association between a lactate clearance of less than 32.8% within 12 h and an increased risk of in-hospital mortality [34,35]. In our study, lactate clearance was 40.69%, with higher levels observed in the early initiation group than in the late initiation group (74.82% vs. 2.86%; *p* < 0.001). The univariable analysis further demonstrated a significant association between lactate clearance and in-hospital mortality (*p* = 0.008). This suggests that early administration of PMX-DHP may contribute to improved lactate clearance, consequently reducing in-hospital mortality.

Through the modulation of activated mononuclear cells and neutrophils, PMX-DHP improves pulmonary oxygenation by suppressing inflammatory mediators and systemic inflammation [36]. In this study, the P/F ratio increased post-PMX-DHP treatment in both groups, with the early initiation group showing a marginally larger increase compared with the late initiation group. This difference, however, was not statistically significant (85.10 ± 189.16 vs. 25.48 ± 129.19; *p* = 0.237). The P/F ratio was assessed 24 h after PMX-DHP in the current study; this fact might explain the differences between our findings and those of previous studies, where the target was acute respiratory distress syndrome or acute lung injury and the P/F ratio was evaluated 72–96 h after PMX-DHP [10,22,36].

Conventionally, the duration of PMX-DHP treatment is 2 h; however, recent studies have reported that, in the event of endotoxin adsorption saturation, extending its duration to >2 h could enhance pulmonary oxygenation and hemodynamics, thereby reducing 28-day mortality. Moreover, a 12 h treatment period may be more effective than a 2 h treatment period in improving mean blood pressures, vasopressor dependency indexes, inotropic scores, serum lactate levels, and the P/F ratio [37,38,39]. Therefore, in our study, PMX-DHP treatment was conducted for 12 h as one cycle. If, after completing one cycle, the vasopressor requirement remained high, indicating anticipated organ failure, an additional treatment cycle was administered. However, no significant correlation was found between treatment frequency and in-hospital mortality (*p* = 0.280).

The most common adverse effects of PMX-DHP are thrombocytopenia, transient hypotension, and allergic reactions [40]. Accordingly, the platelet count after hemoperfusion in the current study decreased in both the early and late initiation groups. The early initiation group showed a more significant reduction in platelet count compared to the late initiation group, but this decrease in platelet count was not associated with in-hospital mortality. This lack of significance in the early initiation group compared with that in the late initiation group may be attributed to the relatively lower amount of platelet transfusion administered in the early initiation group. Notably, our patients did not exhibit transient hypotension or allergic reactions.

This study has some limitations. First, it was a single-center, retrospective study with a small sample size, which limits the generalizability of our findings. Second, we focused on patients with refractory septic shock without initial measurements of endotoxin levels, which limited our analysis of endotoxemia-targeted PMX-DHP treatment effects. However, PMX-DHP is known to induce absorption of endogenous cannabinoids, activated neutrophils, and monocytes, modify monocyte surface marker expression, and be an effective treatment option for Gram-positive bacteremia [36,41]. We can, therefore, infer that PMX-DHP provides beneficial effects for both Gram-negative and Gram-positive bacteremia. Third, in our study, source control was defined as draining an abscess, debriding infected necrotic tissue, removing a potentially infected device, or definitively controlling a source of ongoing microbial contamination. Appropriate source control is a key principle in the management of sepsis and septic shock [4]. Accordingly, we implemented source control in patients when feasible through either surgical or non-surgical interventions (such as drainage of infected fluid or removal of infected devices or foreign bodies). For patients with infections (e.g., respiratory infection, bloodstream infection) in whom the source could not be eliminated through surgical or non-surgical interventions, source control was not performed. Therefore, source control was performed in only 56% of the overall patient population. Fourth, the patients included in this study were those able to afford the treatment cost, which was approximately USD 4000 for one cycle of PMX-DHP in Korea. Given these financial constraints, we could not include all patients presumed to respond favorably to the treatment. Consequently, the selection of participants considered the financial capability and feasibility for the patients’ caregivers. Finally, causes of late initiation also included a prolonged decision-making process by the primary medical department, given that PMX-DHP is not yet considered a standard treatment at the institution conducting the research and delays in determining treatment plans may have played a role in time of initiation.

Despite these limitations, to the best of our knowledge, this is the first study to assess the effects of PMX-DHP in patients with cancer with refractory septic shock, and our findings confirm that the early initiation of PMX-DHP, compared with its late initiation, has a more significant impact on lactate clearance and can reduce in-hospital mortality. Additionally, the application of PMX-DHP decreases multi-organ failure, subsequently lowering in-hospital mortality. In the future, a multicenter, large-scale, randomized, controlled study should be conducted to determine the optimal initiation time.

## 5. Conclusions

In our sample of cancer patients with refractory septic shock, early initiation of PMX-DHP was effective in reducing in-hospital mortality and enhancing lactate clearance. Based on these findings, the early application of PMX-DHP treatment in patients with refractory septic shock can enhance patient outcomes. The optimal initiation time should be determined in a follow-up study.

## Figures and Tables

**Table 1:** Baseline characteristics of the early and late initiation groups.

	Total (*n* = 46)	Early Initiation (*n* = 17)	Late Initiation (*n* = 29)	*p*-Value
Sex				
Female	26 (54.2%)	9 (52.94%)	17 (58.62%)	0.947
Age, years	64.78 ± 11.90	63.18 ± 12.47	65.72 ± 11.67	0.490
Underlying disease				
Diabetes mellitus	14 (29%)	6 (35.29%)	8 (27.59%)	0.829
Hypertension	22 (47.83%)	5 (29.41%)	17(58.62%)	0.108
Cardiovascular disease	6 (13.04%)	1 (5.88%)	5 (17.24%)	0.390
Chronic kidney disease	2 (4.35%)	0	2 (6.90%)	0.524
COPD	1 (2.17%)	0	1 (3.45%)	1.000
Type of cancer				
Genitourinary	25 (54.35%)	10 (58.82%)	15 (51.72%)	0.453
Abdominal	14 (30.43%)	6 (35.29%)	8 (27.59%)	
Others	7 (15.22%)	1 (5.88%)	6 (20.69%)	
APACHE III score	93.54 ± 28.89	92.12 ± 28.98	94.38 ± 29.32	0.801
SOFA score	15.34 ± 3.34	14.88 ± 3.22	15.62 ± 3.44	0.476
No. of PMX-DHP treatments				
1	39 (84.78%)	13 (76.47%)	26 (89.66%)	0.397
2	7 (15.22%)	4 (23.53%)	3 (10.34%)	
PMX-DHP duration (h)	12 (2.08–68.83)	12 (9.58–48)	12 (2.08–68.83)	0.212
Time to PMX-DHP (h)	5 (0.6–27)	2.12 (0.6–3)	6.92 (3.22–27)	<0.001
Primary infection site				
Abdominal	32 (69.56%)	15 (88.24%)	17 (58.62%)	0.076
Non-abdominal	15 (32.61%)	2 (11.76%)	13 (44.83%)	
Laboratory data				
White blood cell count (10^3^/μL)	2.74 (0.01–33.16)	2.77 (0.01–32.14)	2.70 (0.06–33.16)	0.741
Neutropenia (ANC < 1500/μL)	21 (45.65%)	7 (41.18%)	14 (48.28%)	0.873
Hemoglobin level (g/dL)	9.65 (7.2–17.8)	9.90 (7.2–15)	9.60 (8–17.8)	0.964
Platelet count (10^3^/μL)	103 (2–352)	113.00 (15–352)	88.00 (2–235)	0.041
C-reactive protein level (mg/dL)	16.07 (1.08–36.95)	14.82 (1.08–36.95)	15.09 (1.08–30.54)	0.914
Lactic acid level (mg/dL)	74.9 (13.6–304.3)	67.20 (26.5–215.2)	84.50 (13.6–304.3)	0.573
INR	1.65 (1.01–6.11)	1.74 (1.01–4.79)	1.63 (1.18–6.11)	0.750
P/F ratio	137.75 (39.9–524)	156.00 (58.9–417.86)	135.67 (39.9–524)	0.685
Blood culture positive	41 (89.13%)	16 (94.12%)	25 (86.21%)	0.637
Gram-positive	15 (32.61%)	6 (35.29%)	9 (31.03%)	>0.999
Gram-negative	31 (67.39%)	12 (70.59%)	19 (65.52%)	0.977
VIS*	242.50 (12–913)	236 (14–736)	246 (12–913)	0.973
Transfusion				
Red blood cells (packs)	2.50 ± 2.51	2.24 ± 2.52	2.62 ± 2.54	0.675
Plateletpheresis (packs)	1.26 ± 1.50	1 ± 1.32	1.41 ± 1.59	0.371
Fresh frozen plasma (packs)	4.87 ± 6.20	5.24 ± 5.93	4.66 ± 6.45	0.763
Source control	26 (56%)	13 (76.49%)	13 (44.83%)	0.075
Operation	17 (36.96%)	8 (47.06%)	9 (31.03%)	0.441
Interventional	12 (26.09)	6 (35.29%)	6 (20.69%)	0.314
Mechanical ventilation	42 (91.30%)	17 (100%)	25 (86.21%)	0.281
Duration (days)	5.83 ± 4.20	4.48 ± 3.51	7.58 ± 4.55	0.080
CRRT	37 (80.43%)	12 (70.59%)	25 (86.21%)	0.258
Mortality	24 (52.17%)	4 (23.53%)	20 (68.97%)	0.008

VIS*: vasoactive inotrope score = (dopamine dose (µg/kg/min) + dobutamine dose (µg/kg/min) + 100 × epinephrine dose (µg/kg/min) + 10 × milrinone dose (µg/kg/min) + 10,000 × vasopressin dose (units/kg/min) + 100 × norepinephrine dose (µg/kg/min). COPD: chronic obstructive pulmonary disease; APACHE III: Acute Physiology and Chronic Health Evaluation III; SOFA: sequential organ failure assessment; PMX-DHP: polymyxin B-immobilized fiber column direct hemoperfusion; ANC: absolute neutrophil count; INR: international normalized ratio; P/F ratio: ratio of arterial oxygen partial pressure to fraction of inspired oxygen; CRRT: continuous renal replacement therapy.

**Table 2:** Comparative analysis of clinical parameters before and after PMX-DHP: early vs. late initiation.

	Before PMX-DHP	After PMX-DHP	*p*-Value
Early initiation			
Hemoglobin level (g/dL)	9.90	9.100	0.155
Platelet count (10^3^/μL)	113.0	73.50	<0.001
C-reactive protein level (mg/dL)	16.21	18.79	0.016
Lactic acid level (mg/dL)	67.20	35.10	0.322
INR	1.740	1.750	0.322
P/F ratio	156.0	260.8	0.130
SOFA score	15.00	10.00	0.024
VIS*	236.0	12.5	0.011
Late initiation			
Hemoglobin level (g/dL)	9.60	9.000	0.017
Platelet count (10^3^/μL)	88.00	57.00	0.115
C-reactive protein level (mg/dL)	15.92	14.04	0.687
Lactic acid level (mg/dL)	84.5	67.95	0.345
INR	1.630	1.875	0.052
P/F ratio	135.7	240.0	0.458
SOFA score	16.00	13.00	0.009
VIS*	246.0	18.0	<0.001

VIS*: vasoactive inotrope score = (dopamine dose (µg/kg/min) + dobutamine dose (µg/kg/min) + 100 × epinephrine dose (µg/kg/min) + 10 × milrinone dose (µg/kg/min) + 10,000 × vasopressin dose (units/kg/min) + 100 × norepinephrine dose (µg/kg/min). SOFA: sequential organ failure assessment; PMX-DHP: polymyxin B-immobilized fiber column direct hemoperfusion; INR: international normalized ratio; P/F ratio: ratio of arterial oxygen partial pressure to fraction of inspired oxygen.

**Table 3:** Comparison of clinical parameters before and after PMX-DHP: total cohort vs. early and late initiation subgroups.

	Total (*n* = 46)	Early Initiation (*n* = 17)	Late Initiation (*n* = 29)	*p*-Value
Hemoglobin level (g/dL)	−0.80 (−7.8–4.7)	−0.70 (−6.0–1.9)	−0.80 (−7.8–4.7)	0.759
Platelet count (10^3^/μL)	−27(−209–76)	−77.0 (−209–20)	−16.0 (−208–76)	0.017
C-reactive protein level (mg/dL)	2.240 (−12.76–23.97)	1.50 (−2.2–23.97)	−0.24 (−12.76–18.48)	0.025
Lactate clearance (%)	40.69 (−527.25–94.62)	74.82 (−22.92–94.62)	2.86 (−527.25–72.43)	<0.001
INR	0.21 (−2.33–1.77)	0.14 (−0.6–1.77)	0.26 (−2.33–1.28)	0.720
P/F ratio	48.75 ± 155.88	85.10 ± 189.16	25.48 ± 129.19	0.237
SOFA score	−3.17 ± 4.98	−4.00 ± 5.87	−2.64 ± 4.37	0.400
VIS*	−124 (−731–498)	−213 (−731–498)	−108 (−484–441)	0.575

VIS*: vasoactive inotrope score = (dopamine dose (µg/kg/min) + dobutamine dose (µg/kg/min) + 100 × epinephrine dose (µg/kg/min) + 10 × milrinone dose (µg/kg/min) + 10,000 × vasopressin dose (units/kg/min) + 100 × norepinephrine dose (µg/kg/min). SOFA: sequential organ failure assessment; PMX-DHP: polymyxin B-immobilized fiber column direct hemoperfusion; INR: international normalized ratio; P/F ratio: ratio of arterial oxygen partial pressure to fraction of inspired oxygen.

**Table 4:** Univariable and multivariable analyses of in-hospital mortality.

	Univariable Analysis		Multivariable Analysis	*p*-Value
	OR (95% CI)	*p*-Value	OR (95% CI)	
Sex (Female)	1.167 (0.361–3.795)	0.796		
Age	0.986 (0.935–1.036)	0.570		
Underlying disease				
Diabetes mellitus	3.214 (0.874–13.785)	0.091		
Hypertension	1.200 (0.375–3.880)	0.758		
Cardiovascular disease	0.905 (0.151–5.409)	0.909		
APACHE III score	1.011 (0.991–1.034)	0.288		
SOFA score	1.148 (0.959–1.397)	0.144		
Primary infection site				
Abdominal	0.750 (0.204–2.649)	0.656		
Positive blood culture	1.736 (0.261–14.257)	0.567		
Gram-positive	1.600 (0.463–5.821)	0.461		
Gram-negative	0.933 (0.266–3.230)	0.913		
Source control	0.818(0.250–2.638)	0.737		
Operation	0.722 (0.213–2.405)	0.595		
Interventional	1.400 (0.373–5.570)	0.620		
No. of PMX-DHP treatment	2.632 (0.500–19.953)	0.280		
PMX-DHP duration (h)	1.019(0.972–1.080)	0.455		
Time to PMX-DHP (Early initiation)	0.139 (0.032–0.509)	0.005	0.033 (0.002–0.462)	0.011
VIS*	1.012 (0.998–1.004)	0.413		
Variation of values				
Platelet count	0.999 (0.990–1.010)	0.972		
C-reactive protein level	0.774 (0.610–0.902)	0.008		
Lactate clearance	0.990 (0.980–0.997)	0.018		
SOFA score	1.460 (1.197–1.931)	0.001	1.616 (1.204–2.170)	0.001
*VIS* variation	1.007 (1.002–1.013)	0.012		
Mechanical ventilator duration (days)	0.983 (0.848–1.140)	0.817		

VIS*: vasoactive inotrope score = (dopamine dose (µg/kg/min) + dobutamine dose (µg/kg/min) + 100 × epinephrine dose (µg/kg/min) + 10 × milrinone dose (µg/kg/min) + 10,000 × vasopressin dose (units/kg/min) + 100 × norepinephrine dose (µg/kg/min). OR: odds ratio; CI: confidence interval; SOFA: sequential organ failure assessment; PMX-DHP: polymyxin B-immobilized fiber column direct hemoperfusion; APACHE III: Acute Physiology and Chronic Health Evaluation III.

## Data Availability

The datasets used and/or analyzed during the current study are available from the corresponding author on reasonable request.
